# Color Changes in Artificially Induced Incipient Caries after Photodynamic Therapy with Different Concentrations of Methylene Blue and Toluidine Blue and Irrigation with Water and Hypochlorite

**DOI:** 10.1155/2024/6624453

**Published:** 2024-08-30

**Authors:** Sedighe Sadat Hashemikamangar, Mohammadreza Khadivi Moghadam, Mahtab Vahedi, Marzieh Rohaninasab, Nasim Chiniforush

**Affiliations:** ^1^ Department of Restorative Dentistry School of Dentistry Tehran University of Medical Sciences, Tehran, Iran; ^2^ Department of Surgical Sciences and Integrated Diagnostics University of Genoa, Viale Benedetto XV, Genoa, Italy

## Abstract

**Aim:**

The aim of this study was to assess the color changes in artificially induced incipient caries after photodynamic therapy (PDT) using different concentrations of methylene blue and toluidine blue, along with irrigation using water and hypochlorite.

**Materials and Methods:**

Forty-two sound human premolar teeth were used to create two artificial incipient carious lesions. One lesion was placed on the buccal surface and the other on the lingual surface. The color of these artificial incipient carious surfaces was determined using the CIE *L* ^*∗*^*a* ^*∗*^*b* ^*∗*^ color system. The teeth were then randomly assigned to 12 groups (*n* = 7) based on the PDT method. These methods included methylene blue with concentrations of 50, 100, and 150 *µ*g/mL, followed by water irrigation, methylene blue with concentrations of 50, 100, and 150 *µ*g/mL, followed by hypochlorite solution irrigation, toluidine blue with concentrations of 50, 100, and 150 *µ*g/mL, followed by water irrigation, and toluidine blue with concentrations of 50, 100, and 150 *µ*g/mL, followed by hypochlorite solution irrigation. The teeth underwent a colorimetry procedure again, and the resulting color changes were calculated. A three-way ANOVA was performed to analyze the effects of laser wavelength, concentration of the light-absorbing material, and irrigation solution on *ΔE*.

**Results:**

The results showed that the color changes caused by toluidine blue photosensitizer at a concentration of 100 *µ*g/mL, with both water and hypochlorite irrigation, were not noticeable to the naked eye (*ΔE*water = 3.04, *ΔE*hypochlorite = 2.00). However, in the other study groups, the color changes were perceptible (*ΔE* > 3.3). There were no significant differences in *ΔE* between different concentrations of methylene blue and toluidine blue when using either water or hypochlorite irrigation (*P*  > 0.05). A significant difference was observed between methylene blue and toluidine blue at a concentration of 100 *µ*g/mL with water irrigation (*P*=0.006). Additionally, a significant difference was found between methylene blue and toluidine blue at a concentration of 100 *µ*g/mL with hypochlorite irrigation (*P*=0.049). However, no significant differences were observed between methylene blue and toluidine blue at other concentrations with either water or hypochlorite irrigation (*P*  > 0.05).

**Conclusion:**

In conclusion, tooth color in teeth with incipient caries did not change significantly after PDT using toluidine blue (the photosensitizer) at a concentration of 100 *µ*g/mL with either water or 1% hypochlorite solution irrigation for 5 s.

## 1. Introduction

Dental caries is one of the most common chronic diseases worldwide [1]. It affects the hard structures of the teeth and is a chronic process that occurs due to the presence and interaction of factors such as microorganisms, diet, and host [2]. The most important factor in the development of dental caries is the interaction between a high-carbohydrate diet and specific bacteria in dental biofilms. These bacteria produce acid by fermenting the carbohydrates, which results in a decrease in the pH of tooth enamel and the dissolution of its minerals [3]. Conservative dentistry aims to use minimally invasive approaches to preserve the health and integrity of dental tissues during restorative, preventive, and remineralization procedures, with minimal intervention [4]. Nonsurgical management or treatment of dental caries aims to prevent the initiation and progression of new carious lesions [5]. This treatment aims to arrest the caries process at a subclinical level or stop its progression at a clinical and radiographic level [6]. The main components of nonsurgical caries management include toothbrushing with fluoride-containing toothpastes, fluoride therapy, the use of calcium phosphate, casein phosphopeptide-amorphous calcium phosphate, silver diamine fluoride, lasers, dietary modifications, and dental hygiene measures [5, 7, 8].

Photodynamic therapy (PDT) consists of three components: a photosensitizer, an activating light, and molecular oxygen. The efficacy of PDT depends on various factors, such as the wavelength and its reaction with the photosensitizer, the output power, the duration of irradiation, the beam diameter, the emission mode (continuous or pulsed), and the beam application mode (focused or nonfocused) [9]. The goal is to achieve the best efficacy in removing all cariogenic bacteria and affected structures while having the least effect on healthy cells [10]. Red light sources, with a wavelength of 630‒700 nm, are commonly used in PDT as activating light because they can effectively penetrate biological tissues [9]. Light-sensitive agents absorb light with the appropriate wavelength, and this absorbed light activates light-sensitive molecules[11], inducing reactions that produce reactive oxygen species (ROS) that destroy target cells. There are two types of ROS produced: one through electron transfer (type I reaction) and another through energy transfer (type II reaction). The transfer of electrons to O_2_ results in the production of superoxide, hydrogen peroxide, and hydroxyl radicals, whereas the transfer of energy to O_2_ leads to the formation of singlet oxygen [9].

Phenothiazine-based dyes, such as toluidine blue and methylene blue, are light-sensitive and commonly used agents in antimicrobial PDT in dentistry, with favorable results [12, 13, 14, 15, 16, 17]. Toluidine blue is composed of hydrophilic, has a hydrophobic component, and has a positive charge and low molecular weight. Its maximum absorption occurs at 620–660 nm [18]. Due to its hydrophilicity, low molecular weight, and positive charge, toluidine blue can penetrate the purine protein channels in the outer membrane of both Gram-negative and Gram-positive bacteria [19]. However, being a color agent, it can cause tooth discoloration [12]. This is a concern for individuals seeking dental treatment, as the appearance of teeth is important for their esthetics and psychological well-being [12, 20]. While the antibacterial effect of antimicrobial PDT has been extensively studied [11, 21, 22, 23, 24, 25], only a few studies have examined the impact of light-sensitive agents on tooth discoloration [12, 20, 26]. Therefore, there is a lack of sufficient data on the removal of methylene blue from tooth structures after antimicrobial PDT, which calls for further evaluations using different colors and concentrations to establish a standard protocol for minimizing tooth discoloration [26]. Furthermore, most previous studies [12, 20, 26] have focused on the discoloration of tooth root canals due to PDT, with no research available on the effects of PDT on enamel discoloration. Hence, the present study aims to evaluate the discoloration of demineralized enamel following PDT using varying concentrations of methylene blue, toluidine blue, and irrigation with water and hypochlorite solution. The null hypothesis is that there is no relationship between PDT using 50–100 and 150 *µ*g/mL of methylene blue and toluidine blue, as well as washing with water or hypochlorite, on the color change of demineralized enamel based on the CIE *L* ^*∗*^*a* ^*∗*^*b* ^*∗*^ system.

## 2. Materials and Methods

### 2.1. Sample Selection

After this in vitro study's protocol was approved by the Ethics Committee (IR.TUMS.DENTISTRY.REC.1400.097) at Tehran University of Medical Sciences, sound human molar teeth, which were extracted for therapeutic reasons, were selected. Teeth with cracks, caries, hypomineralization, and hypoplasia were excluded. All steps of the study were done by two trained operators blindedly. The first and the second operators did not know and contact with each other to minimize the risk of bias. According to the results of the study by Costa et al. [12], using the one-way ANOVA power analysis option of PASS 11 software,*α* = 0.05 and *β* = 0.2, the minimum sample size required for each of the 12 study groups was 7 (total sample size = 84).

### 2.2. Sample Preparation

The teeth were stored in a 0.5% chloramine-T solution for disinfection for 1 week. Before immersion in chloramine, all the calculi were removed from the tooth surfaces using 5–6 periodontal Gracey curettes (Golgran Ind. e Com. de Instrumental Odontológico Ltd.a., Brasília, DF, Brazil). The soft tissues were then removed from the tooth surfaces, and the teeth were polished using a rubber cup and prophylactic paste. Afterward, the teeth were stored in a normal saline solution (0.85% NaCl) until the study began. To prepare for measurements and interventions, all the tooth surfaces were covered with a layer of nail varnish except for a 5 × 5-mm window on the buccal and lingual surfaces, which was cured for 90 s with a UV unit. Finally, a sponge was used to secure the teeth. This stage was conducted by the first investigator.

### 2.3. Initial Colorimetry

The color of the enamel surface was determined using the CIE *L* ^*∗*^*a* ^*∗*^*b* ^*∗*^ color system and the Easyshade colorimetry device. The *L* ^*∗*^ value represents brightness, ranging from 0 to 100, where 0 is black and 100 is white. The *a* ^*∗*^ value indicates the presence of redness (a+) or greenness (a−), while the *b* ^*∗*^ value represents yellowness (b+) or blueness (b−). Before measuring the color of each tooth surface with the Easyshade device, the calibration button was pressed to ensure standardized results, as per the manufacturer's instructions. The colorimetry procedure was conducted under standardized lighting conditions provided by a daylight bulb. Additionally, a white background was used, with a 5 x 5 square cut out to match the size of the instrument's probe. The study samples were marked in a consistent position for all measurements. The colorimetry probe was placed in contact with the tooth surface, maintaining a constant measurement angle by positioning the tip at a right angle to the 5 x 5 cm surface without any nail varnish. This step was performed by the first operator.

### 2.4. pH Cycling and Secondary Colorimetry

Each sample was soaked in a demineralizing solution (NaH_2_PO_2_, 2.2 mM; CaCl_2_, 2.2 mM; acetic acid, 50 mM) for 6 hr (30 mL for each sample) with a pH of 4.8. After that, the samples were placed in a remineralizing solution (potassium dihydrogen phosphate, 0.9 mM; potassium chloride, 130 mM; calcium chloride, 1.5 mM; HEPES solution, 20 mM) for 18 hr. Finally, the samples were rinsed with deionized water for 5 s. The solutions were changed at the end of each cycle. These procedures were repeated daily for 6 days [27]. Then, the color of the demineralized enamel surface was measured in the CIE *L* ^*∗*^*a* ^*∗*^*b* ^*∗*^ system using the Easyshade device. The device was calibrated by the first operator before each measurement procedure.

### 2.5. Study Groups

The samples were assigned to 12 groups (*n* = 7) using the simple randomization method. The groups were determined based on the photosensitizer used, their concentrations, and irrigation with either water or a hypochlorite solution (Table 1). The randomization process was conducted by a second investigator.

### 2.6. PDT

The photosensitizer was applied to the nail varnish-free surface of demineralized enamel using a 30-G syringe. Methylene blue (Nova Teb Pars Co., Iran) was used with a 660-mm red diode laser (Konftec Co., Taiwan) at a power of 150 mW for 1 min (energy density = 15 J/cm^2^, power density = 0.25 W/cm^2^). Afterward, the samples were rinsed with water using a 30-G syringe for 5 s. A 1% hypochlorite solution was used for irrigation, also for 5 s. For toluidine blue (Nova Teb Pars Co., Iran), a 635-nm red diode laser (Konftec Co., Taiwan) was used at a power of 220 mW for 1 min (energy density = 20.4 J/cm^2^, power density = 0.34 W/cm^2^). Again, the samples were rinsed with water for 5 s using a 30-G syringe. A 1% hypochlorite solution was used for irrigation, also for 5 s. The second operator performed all stages of PDT.

### 2.7. Final Colorimetry

Finally, the samples underwent a colorimetry procedure using the Easyshade colorimeter to analyze color changes. The first operator was blinded during the analysis. *ΔE* was determined using the following formula:

(1) $$\begin{array}{c} \Delta E=\left[\left(\Delta L\right)2+\left(\Delta a\right)2+\left(\Delta b\right)2\right]1/2\left(\Delta L=L_{1}-L_{0},\Delta a=a_{1}-a_{0},\text{ and }\Delta b=b_{1}\text{–}b_{0}\right). \end{array}$$

### 2.8. Statistical Analysis

One-way ANOVA was used to compare the raw initial *ΔE* between the 12 study groups. Then, a three-way ANOVA was conducted to evaluate the effect of laser wavelength, photosensitizer concentration, and irrigation solution on *ΔE*. Additionally, Tukey HSD tests were employed to perform pairwise comparisons between the groups.

## 3. Results

**Figure 1:**
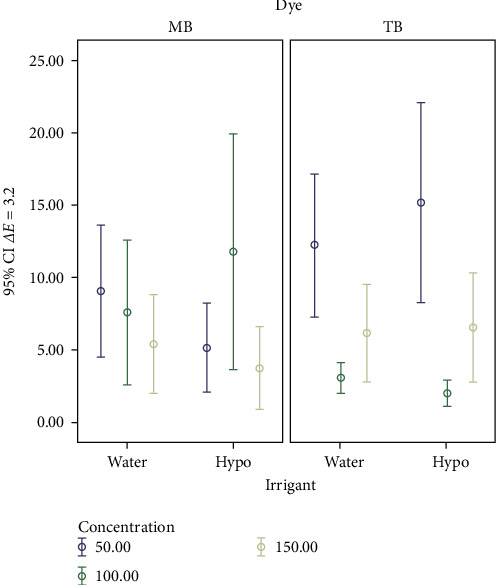
Mean *ΔE* after applying the photosensitizer compared to after inducing artificial incipient caries.

Tables 2, 3, and 4 present *ΔE*, *Δa*, and *Δb* in all the study groups. Intergroup comparisons were conducted using Tukey HSD tests, and the results obtained after PDT and irrigation, compared to after inducing artificial incipient caries, are presented in Tables 5, 6, and 7 (Figure 1). No significant differences in color changes were observed when using methylene blue and irrigating with water at different concentrations (*P*-value ≤0.05). Similarly, no significant differences in color changes were found when irrigating methylene blue with hypochlorite solution, either between the 50 and 100 *µ*g/mL concentrations or between the 50 and 150 *µ*g/mL concentrations. However, a significant difference was observed between the 100 and 150 *µ*g/mL concentrations (*P* ≤ 0.05). Significant differences in color changes were observed when irrigating toluidine blue with water, both between the 50 and 100 *µ*g/mL concentrations (P50-100 *P* ≤ 0.01) and between the 50 and 150 *µ*g/mL concentrations (P50-150 *P* ≤ 0.05). However, no significant difference was found between the 100 and 150 *µ*g/mL concentrations. Similarly, significant differences in color changes were observed when using toluidine blue and irrigating with hypochlorite solution, both between the 50 and 100 *µ*g/mL concentrations (P50-100 *P* ≤ 0.01) and between the 50 and 150 *µ*g/mL concentrations (P50-150 *P* ≤ 0.01). However, no significant difference was found between the 100 and 150 *µ*g/mL concentrations.

According to Table 5, there were no significant differences in color changes with methylene blue between irrigation with water and hypochlorite solution at different concentrations. Furthermore, there were no significant differences in color changes with toluidine blue at different concentrations between irrigation with water and hypochlorite solution.

According to Table 6, there was no significant difference in color changes between methylene blue and toluidine blue with a concentration of 50 *µ*g/mL, regardless of whether they were irrigated with water or with a hypochlorite solution. However, there was a significant difference in color changes between methylene blue and toluidine blue with a concentration of 100 *µ*g/mL, both when irrigated with water and when irrigated with a hypochlorite solution. Similarly, there was no significant difference in color changes between methylene blue and toluidine blue with a concentration of 150 *µ*g/mL, regardless of whether they were irrigated with water (*P*=0.715) or with a hypochlorite solution (*P*=0.155).

## 4. Discussion

The present study evaluated color changes in demineralized enamel after antimicrobial PDT using methylene blue and toluidine blue as photosensitizers. Three different concentrations (50, 100, and 150 *µ*g/mL) were used, and irrigation with water and hypochlorite solution were applied. According to the results, the color changes caused by the photosensitizers were not visible to the human eye, except for the 100 *µ*g/mL concentration of toluidine blue when used with irrigation using water or hypochlorite solution.

In the methylene blue group, with a concentration of 50 *µ*g/mL and irrigation using water or hypochlorite solution, the color change remained in the red spectrum according to the  ^*∗*^*a* parameter, but the intensity of redness increased. Additionally, according to the *b* parameter, it removed some yellow, but the degree of yellowness increased. The color change in these two groups did not revert to the color of the initial carious lesion before the application of the light-sensitive agent. In the methylene blue group, with a concentration of 100 *µ*g/mL and irrigation using water, the color change remained in the red spectrum according to the *a* parameter; however, the intensity of redness decreased. It remained yellow according to the *b* parameter, but the degree of yellowness decreased as well. The color change in this group did not revert to the color of the initial carious lesion before the application of the light-sensitive agent.

In the methylene blue group, with a concentration of 100 *µ*g/mL and irrigation using hypochlorite solution, the color change remained in the red spectrum according to the *a* parameter, and the intensity of redness increased. It remained yellow according to the *b* parameter, and the degree of yellowness increased as well. The color change in this group did not revert to the color of the initial carious lesion before the application of the light-sensitive agent.

In the methylene blue group, with a concentration of 150 *µ*g/mL and irrigation using water or hypochlorite solution, the color change remained in the red spectrum according to the a parameter, and the intensity of redness increased. It remained yellow according to the b parameter, but the degree of yellowness increased as well. The color change in this group did not revert to the color of the initial carious lesion before the application of the light-sensitive agent.

In summary, at a concentration of 100 *µ*g/mL, it was expected that the color change caused by toluidine blue could be reversed by irrigating with water or a hypochlorite solution. However, hypochlorite was found to be superior to water, though the difference was not significant. The only significant difference observed was between methylene blue and toluidine blue at a concentration of 100 *µ*g/mL, with toluidine blue being the superior option. The ability of methylene blue and toluidine blue to penetrate the tooth structure due to their low molecular weight [28] contributes to the color changes they cause.

According to the authors' literature search, there has been no research evaluating tooth enamel discoloration with incipient caries after antimicrobial PDT using photosensitizer materials. Despite the importance of avoiding discoloration in treatments like fluoride therapy and the use of common dyes like methylene blue and toluidine blue in antimicrobial PDT, only a few studies have investigated the effect of photosensitizers on tooth discoloration [12, 20, 26]. Therefore, there is insufficient data available on the removal of dyes used in antimicrobial PDT from the enamel surface, highlighting the need for further studies with different dyes and concentrations to establish a general guideline for their use in antimicrobial PDT [26]. Costa et al. [12] conducted a study to assess the color changes caused by three photosensitizers—toluidine blue, methylene blue, and malachite green—at a concentration of 0.01% after PDT.

The researchers found that the least color changes occurred with antimicrobial PDT using the toluidine blue photosensitizer. There were significant differences observed between toluidine blue and the control groups, with toluidine blue resulting in the least stainability, followed by methylene blue, malachite green, and the control group, respectively. The study concluded that the use of a photosensitizer in PDT as an adjunct treatment for root canal treatment can cause tooth structure discoloration [12]. These findings support the results reported by Ozkocak et al. [28], which also found that toluidine blue caused the least discoloration.

In another study, Figueiredo et al. [20] evaluated tooth discoloration caused by antimicrobial PDT using methylene blue and toluidine blue photosensitizers at a concentration of 0.01% during endodontic treatment. The researchers observed tooth discoloration with both agents, which was then resolved by using Endo-PTC Paste in combination with a 2.5% sodium hypochlorite solution. The study also noted that the tooth discoloration was more pronounced with the use of 0.01% methylene blue compared to 0.01% toluidine blue. This difference may be due to the lower molecular weight of toluidine blue (107.17 g/mL) compared to methylene blue (375.91 g/mL), allowing for greater penetration into the dentinal tubules. Variations in the duration of laser irradiation (5 and 10 min) and the positioning of the photosensitizer may have also contributed to the discrepancy. In the previous study, the photosensitizer was placed inside the root canals near the dentinal tubules, while in the present study, it was placed on demineralized dentin. One limitation of this study was the requirement to locate and collect intact teeth that exhibited no discoloration or deformities and that were roughly equivalent in size and shape. Another limitation was providing diffefent concentrations of the solutions. We did these with more punctuality.

Carvalho et al. [6, 26] and Silva et al. [29] conducted a study to examine the effects of chemical agents on the removal of 0.01% methylene blue after antimicrobial PDT in root canal treatment. The researchers suggested specific protocols to minimize tooth stains and discoloration caused by the photosensitizer after antimicrobial PDT. They found that irrigation with a 2.5% sodium hypochlorite solution in combination with CPT-endo was significantly more effective than using alcohol or a normal saline solution. The results of the present study align with those reported by Carvalho et al. [26] in terms of the impact of hypochlorite solution on stain removal. The researchers also noted the potential significance of chemical interactions between hypochlorite and the photosensitizers in this reaction. This comprehensive study evaluated the efficacy of two photosensitizers, methylene blue and toluidine blue, at concentrations of 50, 100, and 150 *µ*g/mL, while utilizing different irrigation solutions (water and hypochlorite solution). The objective of the study was to determine the optimal concentration and the most effective irrigation solution. Overall, it was found that a concentration of 100 *µ*g/mL of toluidine blue produced superior results in preserving the color of demineralized enamel compared to other concentrations of both toluidine blue and methylene blue. Consequently, it is suggested that a concentration of 100 *µ*g/mL of toluidine blue can serve as an appropriate substitute for methylene blue in antimicrobial PDT. Further research is recommended to explore the tooth discoloration that may occur following antimicrobial PDT using the photosensitizer indocyanine green. This is attributed to its higher molecular weight relative to methylene blue and toluidine blue, as well as its potential limited penetration into tooth structures.

## 5. Conclusion

Within the limitations of this study, preserving the color of a tooth with incipient caries is possible after PDT. This therapy involves using the toluidine blue photosensitizer with a concentration of 100 *µ*g/mL. The tooth is irrigated with either water or a 1% hypochlorite solution for 5 s.

## Figures and Tables

**Table 1:** Study groups.

Group number	Photosensitizer	Irrigating solution
1	50 *μ*g/mL methylene blue	Water
2		Hypochlorite
3	100 *μ*g/mL methylene blue	Water
4		Hypochlorite
5	150 *μ*g/mL methylene blue	Water
6		Hypochlorite
7	50 *μ*g/mL toluidine blue	Water
8		Hypochlorite
9	100 *μ*g/mL toluidine blue	Water
10		Hypochlorite
11	150 *μ*g/mL toluidine blue	Water
12		Hypochlorite

**Table 2:** Mean *ΔE* values of enamel after antimicrobial PDT (with different concentrations of methylene blue).

Irrigating solution	Concentration	Irrigation solution	Color changes	Mean	SD
Methylene blue	50 *μ*g/mL	Water	*ΔE* = 2.1	22.17	7.13
			*ΔE* = 3.1	23.17	4.06
			*ΔE* = 3.2	9.06	4.95
		Hypochlorite	*ΔE* = 2.1	18.07	2.99
			*ΔE* = 3.1	17.21	4.57
			*ΔE* = 3.2	5.12	3.32
	100 *μ*g/mL	Water	*ΔE* = 2.1	15.94	2.59
			*ΔE* = 3.1	15.48	1.26
			*ΔE* = 3.2	7.55	5.39
		Hypochlorite	*ΔE* = 2.1	23.00	4.99
			*ΔE* = 3.1	24.71	7.46
			*ΔE* = 3.2	11.78	8.83
	150 *μ*g/mL	Water	*ΔE* = 2.1	14.48	7.72
			*ΔE* = 3.1	18.35	7.68
			*ΔE* = 3.2	5.37	3.68
		Hypochlorite	*ΔE* = 2.1	12.72	7.71
			*ΔE* = 3.1	14.54	8.02
			*ΔE* = 3.2	3.72	3.07

*ΔE* = 2.1: the color change between the tooth baseline color and its color after creating incipient caries. *ΔE* = 3.1: the color change between the tooth baseline color and its color after antimicrobial photodynamic therapy and irrigation. *ΔE* = 3.2: the color change between the artificial incipient caries and the tooth color after antimicrobial photodynamic therapy and irrigation. *ΔE* < 3.3 is not perceptible by the eye.

**Table 3:** Mean *ΔE* values after PDT (with different concentrations of toluidine blue).

Irrigating solution	Concentration	Irrigation solution	Color changes	Mean	SD
Toluidine blue	50 *μ*g/mL	Water	*ΔE* = 2.1	13.09	3.08
			*ΔE* = 3.1	20.45	2.81
			*ΔE* = 3.2	12.22	5.36
		Hypochlorite	*ΔE* = 2.1	23.70	5.97
			*ΔE* = 3.1	24.91	2.43
			*ΔE* = 3.2	15.18	7.48
	100 *μ*g/mL	Water	*ΔE* = 2.1	20.82	2.81
			*ΔE* = 3.1	21.67	3.43
			*ΔE* = 3.2	3.04	1.14
		Hypochlorite	*ΔE* = 2.1	21.65	8.66
			*ΔE* = 3.1	20.73	8.53
			*ΔE* = 3.2	2.00	0.97
	150 *μ*g/mL	Water	*ΔE* = 2.1	16.33	5.20
			*ΔE* = 3.1	18.17	7.11
			*ΔE* = 3.2	6.17	3.63
		Hypochlorite	*ΔE* = 2.1	19.39	6.18
			*ΔE* = 3.1	14.16	5.41
			*ΔE* = 3.2	6.58	4.07

*ΔE* = 2.1: the color change between the tooth baseline color and its color after creating incipient caries. *ΔE* = 3.1: the color change between the tooth baseline color and its color after antimicrobial photodynamic therapy and irrigation. *ΔE* = 3.2: the color change between the artificial incipient caries and the tooth color after antimicrobial photodynamic therapy and irrigation. *ΔE* < 3.3 is not perceptible by the eye.

**Table 4:** Mean *Δa* and *Δb* after applying the photosensitizer compared to after inducing artificial incipient caries.

Material	Concentration	Irrigant	Color changes	Mean	After caries induced	After irrigation
Methylene blue	50 *μ*g/mL	Water	*Δa*	1.6857	*a*_2_ = 8.614	*a*_3_ = 10.300
		Hypochlorite	*Δa*	0.9000	*a*_2_ = 7.200	*a*_3_ = 8.100
		Water	*Δb*	6.5429	*b*_2_ = 39.329	*b*_3_ = 45.871
		Hypochlorite	*Δb*	1.4714	*b*_2_ = 42.286	*b*_3_ = 43.757
	100 *μ*g/mL	Water	*Δa*	−3.1857	*a*_2_ = 7.957	*a*_3_ = 4.771
		Hypochlorite	*Δa*	1.9143	*a*_2_ = 6.971	*a*_3_ = 8.886
		Water	*Δb*	−5.2857	*b*_2_ = 40.186	*b*_3_ = 34.900
		Hypochlorite	*Δb*	4.9286	*b*_2_ = 38.529	*b*_3_ = 43.457
	150 *μ*g/mL	Water	*Δa*	0.5286	*a*_2_ = 5.971	*a*_3_ = 6.500
		Hypochlorite	*Δa*	1.3571	*a*_2_ = 6.371	*a*_3_ = 7.729
		Water	*Δb*	0.1571	*b*_2_ = 39.057	*b*_3_ = 39.214
		Hypochlorite	*Δb*	1.3714	*b*_2_ = 41.771	*b*_3_ = 43.143

Toluidine blue	50 *μ*g/mL	Water	*Δa*	4.0143	*a*_2_ = 5.000	*a*_3_ = 9.014
		Hypochlorite	*Δa*	4.4429	*a*_2_ = 7.729	*a*_3_ = 12.171
		Water	*Δb*	9.5000	*b*_2_ = 39.857	*b*_3_ = 50.271
		Hypochlorite	*Δb*	10.4143	*b*_2_ = 42.286	*b*_3_ = 43.757
	100 *μ*g/mL	Water	*Δa*	−0.7000	*a*_2_ = 8.700	*a*_3_ = 8.000
		Hypochlorite	*Δa*	0.5714	*a*_2_ = 5.486	*a*_3_ = 6.057
		Water	*Δb*	0.2143	*b*_2_ = 41.143	*b*_3_ = 41.357
		Hypochlorite	*Δb*	−0.2143	*b*_2_ = 35.586	*b*_3_ = 35.371
	150 *μ*g/mL	Water	*Δa*	0.3286	*a*_2_ = 6.129	*a*_3_ = 6.457
		Hypochlorite	*Δa*	−1.1286	a_2_ = 6.514	*a*_3_ = 5.386
		Water	*Δb*	−0.1573	*b*_2_ = 39.357	*b*_3_ = 39.200
		Hypochlorite	*Δb*	−0.8286	*b*_2_ = 38.914	*b*_3_ = 38.086

*a*_2_ = the parameter *a* after inducing artificial incipient caries. *a*_3_ = the parameter *a* after antimicrobial photodynamic therapy and irrigation. *b*_2_ = the parameter *b* after inducing artificial incipient caries. *b*_3_ = the parameter *b* after antimicrobial photodynamic therapy and irrigation. *Δa* = a_3_−a_2_. *Δb* = *b*_3_−*b*_2_.

**Table 5:** Comparison between different concentrations in different groups.

Color changes	Material	Irrigation solution	*P* value
*ΔE* = 3.2	Methylene blue	Water	*P*_50–100_=0.824
			*P*_50–150_=0.325
			*P*_100–150_=0.325
		Hypochlorite	*P*_50–100_=0.103
			*P*_50–150_=0.891
			*P*_100–150_=0.043
	Toluidine blue	Water	*P*_50–100_=0.001
			*P*_50–150_=0.021
			*P*_100–150_=0.295
		Hypochlorite	*P*_50–100_=0.01
			*P*_50–150_=0.012
			*P*_100–150_=0.222

*ΔE* = 3.2: the color change between the artificial incipient caries and the tooth color after antimicrobial photodynamic therapy and irrigation. *P*  < 0.05: there is a significant difference.

**Table 6:** Comparison between different irrigation solutions in different groups.

Color changes	Material	Concentration	Irrigation solution	*P* value
*ΔE* = 3.2	Methylene blue	50	Water	*P*=0.431
			Hypochlorite	
		100	Water	*P*=0.551
			Hypochlorite	
		150	Water	*P*=0.284
			Hypochlorite	
	Toluidine blue	50	Water	*P*=0.588
			Hypochlorite	
		100	Water	*P*=0.991
			Hypochlorite	
		150	Water	*P*=0.446
			Hypochlorite	

*ΔE* = 3.2: the color change between the artificial incipient caries and the tooth color after antimicrobial photodynamic therapy and irrigation. *P*  < 0.05: there is a significant difference.

**Table 7:** Comparison between different materials in different groups.

Color changes	Concentration	Irrigation solution	Material	*P* value
*ΔE* = 3.2	50	Water	Methylene blue	*P*=0.697
			Toluidine blue	
		Hypochlorite	Methylene blue	*P*=0.151
			Toluidine blue	
	100	Water	Methylene blue	*P*=0.006
			Toluidine blue	
		Hypochlorite	Methylene blue	*P*=0.049
			Toluidine blue	
	150	Water	Methylene blue	*P*=0.715
			Toluidine blue	
		Hypochlorite	Methylene blue	*P*=0.155
			Toluidine blue	

*ΔE* = 3.2: the color change between the artificial incipient caries and the tooth color after antimicrobial photodynamic therapy and irrigation. *P*  < 0.05: there is a significant difference.

## Data Availability

The datasets analyzed during this study are not publicly available but are available from the corresponding author upon reasonable request.
